# Initial Management and Recognition of Aortoiliac Occlusive Disease, A Case Report

**DOI:** 10.21980/J87M0Z

**Published:** 2022-01-15

**Authors:** Ashley Hope, Alisa Wray, Graham Stephenson

**Affiliations:** * University of California, Irvine, Department of Emergency Medicine, Orange, CA

## Abstract

Aortoiliac occlusive disease (AOD) is a manifestation of peripheral arterial disease characterized by stenosis or occlusion of the distal aorta and iliac vessels. Advanced disease may present with symptoms of claudication to the buttock and thighs, erectile dysfunction, and absent or diminished femoral pulses bilaterally. Here, we discuss a case of a 71-year-old male who presented with acute bilateral lower extremity pain and weakness. Pulses were undetectable bilaterally at the groin prompting emergent imaging and vascular surgery consultation due to the suspected diagnosis of AOD. The patient was taken for bilateral femoral artery cut downs with embolectomy. The patient made an impressive recovery with preservation of motor function of lower extremities. In this case report we discuss pertinent workup and management of a patient suspected to be suffering from AOD.

**Topics:**

Vascular, arterial thrombosis, limb ischemia, aortoiliac arterial thrombosis, Leriche syndrome, peripheral arterial disease.

[Fig f1-jetem-7-1-v1]

**Figure f1-jetem-7-1-v1:**
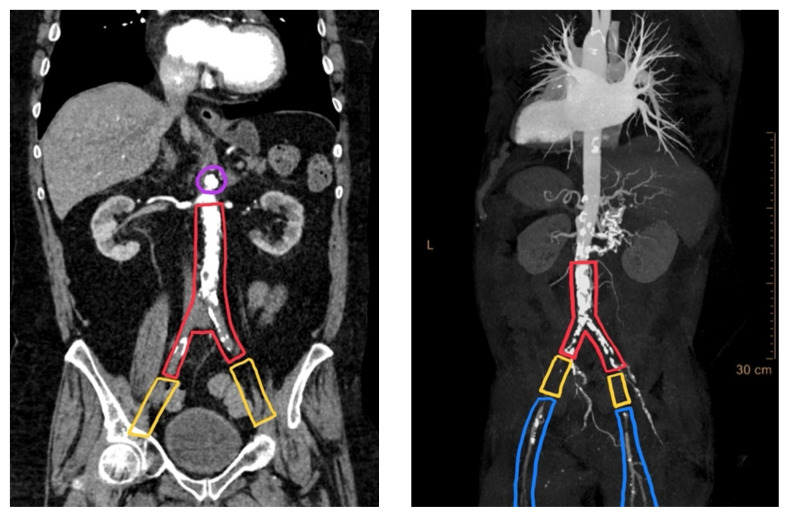
Annotated Axial CT Video Link: https://youtu.be/TYacFMkl-FM Unannotated Axial CT Video Link: https://youtu.be/MkgKogYWUnc

## Brief introduction

Atherosclerosis, or the accumulation of lipid and fibrous material in arterial walls, may lead to arterial insufficiency over time due to vessel stenosis. The non-coronary manifestations of this process are referred to as peripheral arterial disease (PAD).^1^ This is a common condition, estimated to affect 4.3% of adults over 40 in the United States.^2^ An ankle brachial index (ABI) of less than 0.9 is considered diagnostic for PAD.^3^ Predisposing risk factors include hypertension, hypercholesterolemia, diabetes, coronary artery disease, obesity, tobacco use, and advanced age. 4 Although frequently asymptomatic, PAD may clinically present as atypical leg pain, claudication, or chronic poor wound healing of distal extremities.^5^ Severe cases may include ischemic ulcers, gangrene, abscess formation, or even loss of limb.

Symptomatic atherosclerotic disease involves the abdominal and lower extremity arteries in an estimated 42% of cases.^6^ Aortoiliac occlusive disease (AOD), a rare form of PAD, is characterized by disease of the distal aorta and proximal iliac vessels. 4 Classically, AOD will cause symptoms such as claudication of the buttock and thighs, erectile dysfunction, and absent or diminished pulses. 7 Acute changes to symptoms, such as pain or weakness, may indicate thromboembolic events.^3^ For the emergency provider the rapid identification and treatment are imperative to minimize permanent neurovascular injury which can occur in as little as 4 hours. 8 Here, we discuss a patient presentation concerning for AOD, pertinent initial workup, and subsequent management.

## Presenting concerns and clinical findings

A 71-year-old male presented to the emergency department in significant distress with bilateral lower extremity pain, weakness, and sensation changes that abruptly began one hour prior to presentation. His previous medical history was significant for atrial fibrillation on warfarin for anticoagulation, chronic obstructive pulmonary disease, congestive heart failure, as well as hypertension. Three weeks prior to arrival the patient had discontinued his use of oral anticoagulation. Notable vitals included tachycardia to 112, tachypnea measured at 28 respirations per minute, and blood pressure of 152/123. On exam, the patient had mottling of his bilateral lower extremities extending up to his abdomen, ending just inferior to the umbilicus. Motor function of lower extremities was poor, and he was unable to resist gravity. Dorsalis pedis, popliteal, and femoral pulses were absent bilaterally by palpation which was later confirmed with doppler.

## Significant findings

Computerized tomography with angiography (CTA) of the entire aorta demonstrated an occluded distal infrarenal aorta with extension into the bilateral common femoral arteries (red outline), lack of flow through femoral arteries (yellow outline) and trickle flow reconstituted distally consistent with aortoiliac occlusive disease (blue outline). Some small segments of the proximal celiac axis showed signs of occlusion (purple outline). A short segment of non-specific bowel wall thickening, which may have been related to ischemic changes, was also seen (not seen on images). The included coronal slice shows the extent of the bilateral occlusive burden, with three-dimensional reconstruction emphasizing the same findings.

## Patient course

On initial exam the patient was in clear distress and unable to elevate legs against gravity. Vascular examination was remarkable for absent pulses bilaterally at the groin. The patient was promptly taken for CTA of the whole aorta. Imaging suggested occlusion of the distal infrarenal aorta with extension into the bilateral common femoral arteries. Vascular surgery was immediately notified. Pain control with Hydromorphone was administered because the patient was in notable distress. The patient’s blood pressure continued to increase despite pain control to a max of 247/121, which was subsequently managed with an esmolol drip. Significant lab values included lactic acidosis to 6.2 and modest elevation of brain natriuretic peptide (BNP) to 682. Renal function was within normal limits. International normalized ratio (INR) was 1.05. The patient was taken emergently to the operating room for bilateral femoral artery cut downs with embolectomy. He was later admitted to the intensive care unit for blood pressure management and frequent vascular checks. He worked extensively with physical therapy due to continued weakness over the course of his hospitalization. Warfarin was resumed per his previous regimen after an initial heparin bridge. Patient was discharged to a skilled nursing facility and continues to follow in the vascular clinic.

## Discussion

Aortoiliac occlusive disease (AOD) is a subtype of peripheral arterial disease (PAD) characterized by atherosclerosis of the aorta and iliac vessels. Symptoms range from mild claudication with activity to extreme pain, and motor or neurologic deficits. The rapid development of symptoms may support thromboembolic events occluding the distal aorta often precipitated by atrial fibrillation, aortic aneurysms, or dissection.^9^ Leriche Syndrome, a variant of AOD, is classically defined as the concurrent combination of specific symptoms: claudication of the thighs and buttocks, impotence, absent and reduced femoral pulses bilaterally.

Initial evaluation of the suspected patient should confirm the presence or absence of distal and proximal pulses of the lower extremities. If pulses are present, an ankle brachial index (ABI) serves as an important approximation of vessel disease, with a ratio less than 0.9 considered abnormal.^3^ When there is a high degree of suspicion for AOD, imaging of the entire aorta is preferred because aortic dissection or aneurysm can precipitate the occlusion. 9 The severity of vessel stenosis is best measured in the emergency setting by CT angiography. High grade stenosis, eg, greater than 75%, can be stated with a sensitivity of 93% and specificity of 96% when evaluating with CTA.^10^ Evaluating for end organ ischemia should also be considered standard of care in the initial workup. In some cases, paralysis may be seen due to anterior spinal artery ischemia.^11^

Medical management with unfractionated heparin may be beneficial in patients with mild to moderate disease. However, in patients at high-risk for limb ischemia, the preferred treatment is interventional: thrombolysis, percutaneous thrombectomy, or bypass grafting. The patient highlighted in this case report was considered high-risk due to hypertension, tobacco use, advanced age, heart failure, and occlusion of the distal aorta seen on CTA. It is now increasingly common to perform endovascular therapies since they have comparable secondary patency rates, are associated with shorter hospital stays, and are performed at lower costs with the expense of potentially inferior durability. 4, 7, 12, 13 Surgical treatment has favorable outcomes, with overall primary patency rates of over 77% and limb salvage rates of over 91% after ten years, but with higher risk of operative morbidity.^14^

This report provides an important overview of the initial evaluation and management of aortoiliac occlusive disease. This case is of particular interest due to the severity of occlusion evidenced by impressive imaging, acute change to symptoms in the setting of discontinuing anticoagulation, and remarkable physical exam of complete absence of peripheral pulses of the lower extremities. The favorable outcome for this patient was predicated on a thorough vascular exam, appropriate imaging given the clinical concern, and expedited specialty intervention.

## Supplementary Information








