# Supplementary figures and images for: Initial Management and Recognition of Aortoiliac Occlusive Disease, A Case Report
Source: J Educ Teach Emerg Med. 2022 Jan 15;7(1):V1–4. doi: 10.21980/J87M0Z (PMC10358871; doi:10.21980/J87M0Z)

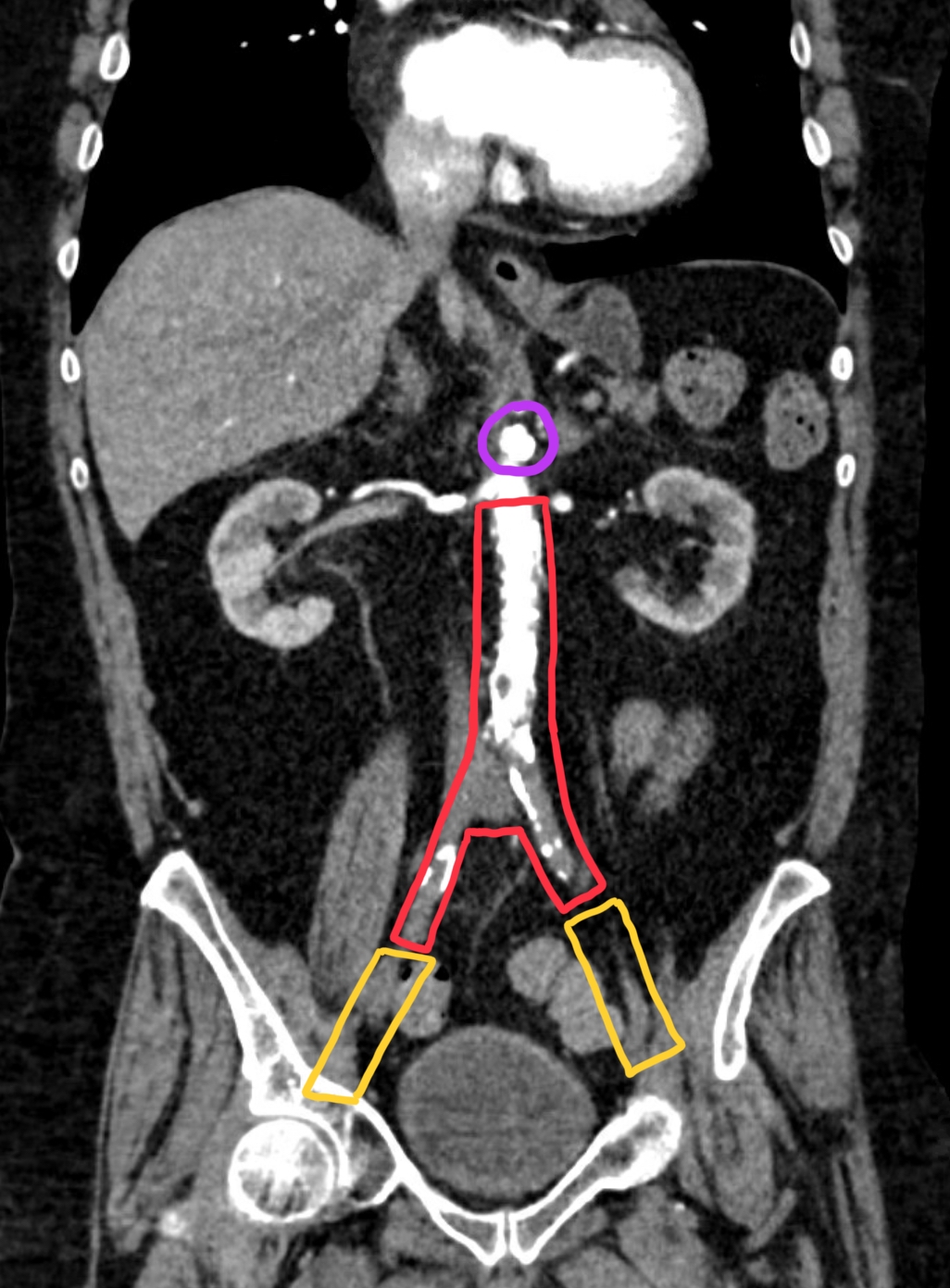

Supplement: Supplementary file 1 [file JETem-7-1-V1-supp1.jpg]

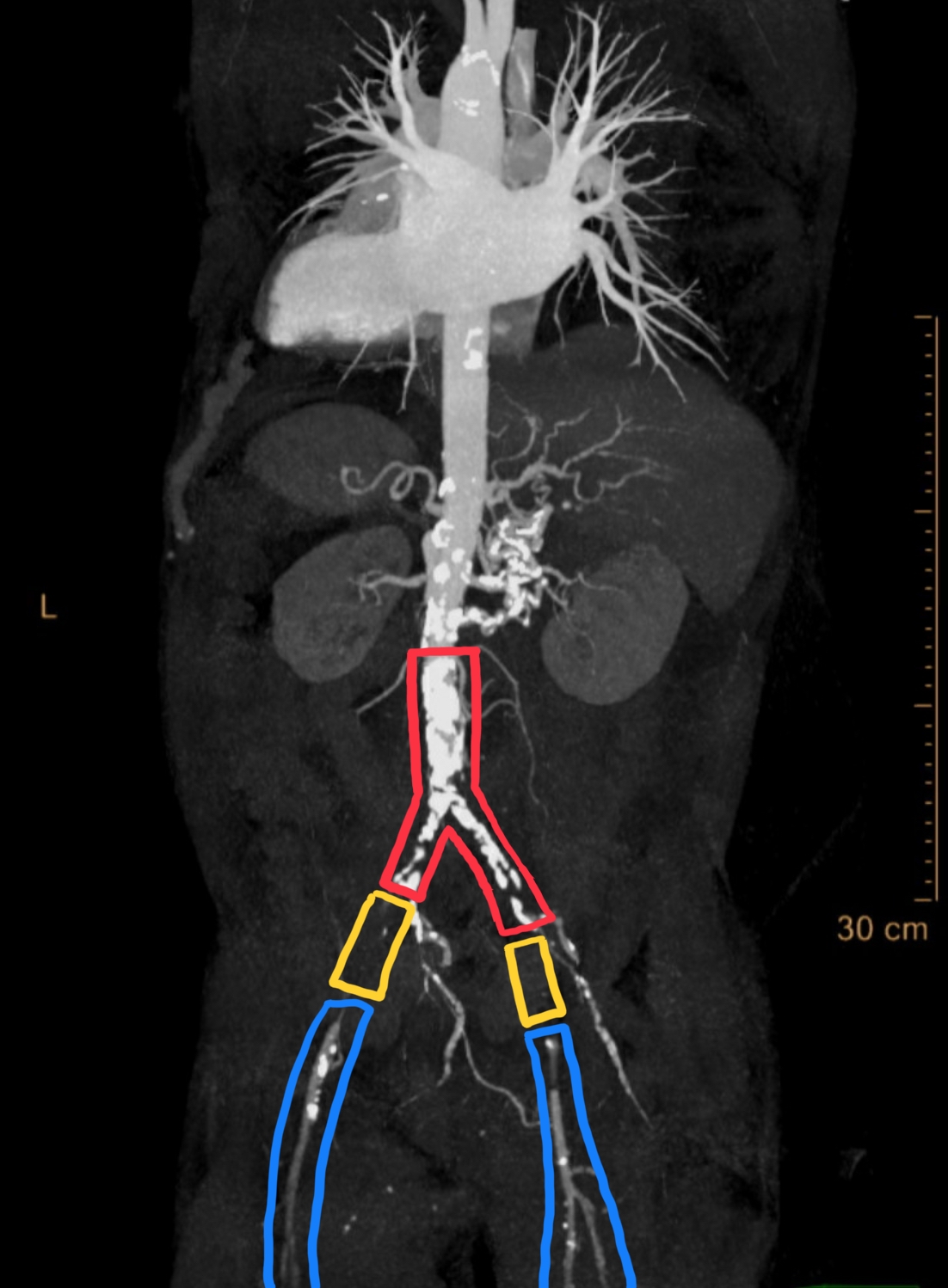

Supplement: Supplementary file 3 [file JETem-7-1-V1-supp2.jpg]
